# Thrombosis: Grand Challenges Ahead!

**DOI:** 10.3389/fcvm.2021.637005

**Published:** 2021-05-04

**Authors:** Hugo ten Cate

**Affiliations:** ^1^Thrombosis Expertise Center, Cardiovascular Research Institute Maastricht, Maastricht University Medical Center, Maastricht, Netherlands; ^2^Center for Thrombosis and Haemostasis, University Medical Center of Gutenberg University, Mainz, Germany

**Keywords:** thrombosis, anticoagulant (MeSH), coagulation factor, protease activated receptor, bleeding

Thrombosis is the formation of a blood clot that partially or completely hinders blood flow in one or more blood vessels, either in the venous or arterial circulation. Thrombosis is the immediate trigger of a number of acute cardiovascular diseases (CVD), including myocardial infarction, ischemic stroke, and venous thromboembolism (VTE), which are all leading causes of mortality and long-term morbidity, affecting men and women of all ethnicities all over the world. In 2016, an estimated 17.9 million people died from CVD, representing 31% of all global deaths; of these deaths, 85% were due to myocardial infarction and stroke, half of which due to thromboembolic brain injury^1^,^1^. The number of VTE events is less accurately monitored, but estimates included 684,000 deep vein thrombosis (DVT) events, 435,000 pulmonary embolism (PE) events, and a total of about 543,000 VTE-related deaths among the population of the European Union, of ~450 million total subjects in 2004 (1).

In all these acute conditions, thrombosis is a *localized* problem, causing ischemia in the downstream vascular bed in case of arterial thrombosis, and impaired return of blood flow to the heart, with a risk of thromboembolic organ injury in the case of VTE, respectively. Thrombosis can also be a more *systemic or combined local-systemic* phenomenon and the current Covid-19 pandemic introduces a dramatic example of systemic hypercoagulability and localized thrombosis, associated with SARS-CoV-2 virus pneumonia (Covid-19). The latter type of systemic coagulopathy, with both microvascular thrombo-inflammation and large vessel thrombosis (and/or VTE), was initially regarded as disseminated intravascular coagulation (DIC), later thrombotic microangiopathy, both attempts to describe and define the specific type of thrombotic tendency (2, 3). Thrombotic thrombocytopenic purpura (4) and heparin induced thrombocytopenia (5) are yet other systemic thrombotic phenomena.

Essentially, thrombosis is an excess response-to-injury, meant to arrest bleeding from a damaged vascular bed, in case of trauma; or, to respond to an infectious attack, involving perturbation of the vascular endothelium by inflammatory cells and platelets (6), secreted inflammatory mediators and generation of extracellular vesicles, challenging the normal anticoagulant endothelial function to turn into a more prothrombotic phenotype (7–10). The latter type of immune mediated thrombosis is also referred to as immunothrombosis (11), but DIC and other systemic coagulopathies also qualify as immune mediated manifestations of thrombo-inflammatory reactions. Whereas, “thrombosis” as a result of an exaggerated response-to-injury is still targeted against *exogenous* elements (traumatic, pathogens, particulate matter) in case of recognizing “self” as “foreign” thrombosis can also be part of autoimmune pathologies like antiphospholipid syndrome (APS) (12). Whatever the cause, thrombosis is either directly harmful, due to vascular occlusion, or indicative of an increased likelihood of dying, in conjunction with severe underlying diseases like cancer (13), or sepsis (DIC also referred to as “death is coming”) (14).

*Primary* thrombosis prevention would be theoretically attractive, but the options are limited and not without risk, because like with the immune system, it can harm and inhibit the coagulation system, and impaired coagulation results in a bleeding tendency. Interestingly, clinical evidence suggests that an increased risk for thrombosis, such as that associated with the carriership of the factor V Leiden mutation, might alter susceptibility for specific infections, which may illustrate a role for coagulation in host defense (15). Experimental studies support a protective effect for factor V Leiden in pneumonia (16). On the other hand, protein C deficiency aggravated polymicrobial sepsis in mice (17), showing the complexity of such risk associations linked to specific coagulation factors, i.e., factor V and protein C (linked via the proteolytic effect of activated protein C on factor Va). A related issue is whether the use of anticoagulant agents would alter susceptibility to infection and this issue has become highly relevant in the current Covid-19 pandemic. Although we are awaiting stronger evidence, recent data suggest that anticoagulation use may protect against mortality related to Covid-19 (18) but in very high-risk patients this protective effect is not yet evident (19). These issues are exciting from an etiological perspective but practical management does not yet take into account thrombophilia traits to initiate thrombosis prophylaxis. The primary prevention of arterial thrombosis is limited to patients with symptomatic atherosclerosis, whereas the use of aspirin in subjects without vascular disease is only considered in those at fairly high risk of CV events, e.g., diabetics. For VTE, primary prevention is limited to high-risk situations, e.g., major surgery and major medical illnesses like Covid-19, or in specific malignancies (multiple myeloma) where chemotherapy brings additional thrombosis risk.

Unfortunately, in many cases, a first thrombosis cannot be properly prevented, and management focuses on optimal *secondary* prevention. In the case of atherothrombosis (thrombosis associated with atherosclerosis, like in myocardial infarction), this means lifelong antiplatelet therapy (APT) with aspirin or a P2Y12 inhibitor or both. In other settings with high risk of arterial thrombosis, like in most patients with atrial fibrillation (AF), but also in all patients with VTE, oral anticoagulation for a limited time, or lifelong (AF) administration, is indicated. These indications concern millions of subjects worldwide. In VTE, secondary prevention also tends to become lifelong in patients with unprovoked VTE.

Based on the above sketched intensive interactions between blood coagulation and cardiovascular and infectious diseases, emerging in a substantial fraction of the global population, it is both timely and important to address a number of specific challenges in future research.

A **first grand challenge** is the substantial risk of major bleeding including potentially fatal bleeding (e.g., intracranial) imposed on all subjects (millions worldwide) that require long-term oral anticoagulation for prevention of (recurrent) thrombosis. A feasible strategy may be to select the optimal oral anticoagulant based on clinical characteristics (e.g., age, renal function, presence of cancer or not) in conjunction with individual biomarker profiles, supporting such clinical decision making (20–22). A second strategy is to develop antithrombotic agents with a better efficacy safety profile and this approach is beginning to take shape in this era. It will be exciting to see whether it is possible to design drugs that target specific points in the hemostatic mechanism that protect against thrombosis, without causing major bleeding (23). Drugs aimed at factor XIa (24) or XIIa may, by acting just outside core enzymes in the coagulation cascade, theoretically provide safer agents, but clinical studies need to corroborate this. “New” targets including kallikrein (25) or PAR-4 on platelets (26) are just a few examples of epitopes that may be blocked to prevent thrombosis, at a lower risk of bleeding.

A **second challenge** is to determine whether *dampening* the coagulation cascade by the above strategies, could pave the way for primary prevention in *more* subjects, also those at risk of atherothrombosis. This should preferably be done in a tailored manner, i.e., taking into account the patient's ambient level of coagulation activity including knowledge of the relevant determinants of this coagulation profile (27). For instance, ambient coagulation may be characterized by hyperreactivity of platelets in one person or by changes in the plasmatic system in the other. In addition, variation in fibrinolytic activity may have consequences for the individual's risk of thrombosis. The net hypercoagulability may require antiplatelet therapy (APT) in one and anticoagulants, or both, in the other person. The combination of low dose rivaroxaban and aspirin is the first and most promising example of combined treatment to further reduce the risk of atherothrombosis (28). Potentially, comparable regimens would work in patients with other thrombotic disorders including those with combined arterial and venous risk factors.

Dampening ambient coagulation activity has similarities with attempts to dampen chronic inflammation. In a recent webinar on the occasion of the American Heart Association's annual meeting, Dr. Paul Ridker discussed the use of tailored anti-inflammatory treatment to dampen the inflammasome in the future, in particular, interleukin (Il)-1 beta and Il-6, as key cytokines (29). He outlined a near future of patient and biomarker tailored modification of the inflammasome to reverse residual cardiovascular (CV) risk (after having implemented management of all conventional CV risk factors like cholesterol and hypertension). For inflammation, this approach finds strong support in the data from Jupiter (stratifying on CRP), Cantos (blocking Il-1beta), and most recent studies with colchicine (30). The idea of similarly using (combined) antithrombotic drugs to also diminish the “thrombo” element of thrombo-inflammatory vascular disease by improving vascular “health” is a tempting and major challenge for the years ahead (27).

A **third challenge** concerns the optimization of the use of *laboratory methods* to improve the efficacy to safety ratio of antithrombotic medication in the cardiovascular arena. Some examples include recent studies that yielded quite impressive data regarding the genotype based, selective use of the platelet inhibitor clopidogrel in place of more potent drugs of the same class of P2Y12 inhibitors, in patients with sufficient metabolizing capacity for the prodrug clopidogrel that required a percutaneous coronary intervention (PCI) and stenting: use of the generic, hence cheaper, clopidogrel was non-inferior as compared to the standard arm, while there was a reduction in bleeding in those treated with clopidogrel (all on top of aspirin) (31). While two other studies either did not or partially supported the advantages of genotyped guided P2Y12 inhibition (32, 33). Data suggest that laboratory supported approaches including genotyping, may potentially finetune antithrombotic management. The use of platelet function testing may find a place in the safer management of patients at very high bleeding risk, i.e., frail elderly people (34).

Another example is the antithrombotic management of patients with thrombosis in conjunction with cancer and bleeding enhancing risk factors like thrombocytopenia, limiting adequate anticoagulant treatment due to bleeding risk (35). In the future, the use of tests like thrombin generation in plasma, with or without platelets, may be useful to better estimate the true clotting potential and the impact of a relative shortage of platelets (36). Moreover, the clinical application of assays that probe the function of platelets in a flow-based vessel-on-a-chip model system, could provide a valuable diagnostic addition to determine the individual's hemostatic capacity, rather than just the platelet number, an unreliable biomarker for bleeding risk (37).

A third example of where laboratory guided therapeutic management could become relevant is in the use of integral laboratory assays like thrombin generation (38), viscoelastic assays like Rotem, TEG, or other methods for studying fibrin clot formation and lysis, preferably in a point of care (POC) application (39). Although viscoelastic assays are increasingly applied in peri-operative and other high risk bleeding settings to guide transfusion management (40), such assays in conjunction with platelet function assays may help to guide prohemostatic dosing [e.g., the use of antidotes in patients bleeding on oral anticoagulants (41)] but also to optimize anticoagulant therapy. An example of the latter is the management of unfractionated heparin in extracorporeal circuits, in which the occurrence of “heparin resistance” can pose substantial management problems as now seen in severe cases of Covid-19 (42).

A **fourth challenge** is to elucidate the different *mechanisms* that underlie specific types of thrombosis. One of the current areas of interest concerns pulmonary thromboembolism. Traditionally, PE is regarded to be a consequence of venous thrombosis at distant sites, most often in the leg/pelvic veins. However, PE also occurs in the absence of overt thrombosis (isolated PE) and may occur *in situ*, as documented in Covid-19 patients. In Covid-19 two pathways may be operational. The first is *in situ* thrombosis resulting from progressive pulmonary infection and thrombo-inflammation due to a microvascular coagulopathy, in a “dose dependent” manner (the worse the disease, the more thrombosis). The second is due to “conventional” embolism from a distant site of venous thrombosis (43). These are clinically relevant for at least two reasons. First, *pulmonary thrombosis* forms in pulmonary arteries, and risk factors may be distinctly different from those that affect VTE. Second, isolated PE (or perhaps better pulmonary thrombosis) appears to be associated more strongly with arterial cardiovascular disease risk factors than isolated venous thrombosis (44). Thus, vascular bed and risk factor specific conditions may determine the type of thrombosis and should perhaps influence treatment and secondary prevention in different ways than currently employed. This perspective also fuels an older discussion on overlapping mechanisms between arterial and venous thrombosis (45).

**Fifth**, we are on the verge of better understanding the ways that selective proteases like thrombin and factor Xa direct processes of thrombo-inflammation and the impact that this crosstalk may have in several complex CVD (such as atherosclerosis, atrial fibrillation, ischemic stroke, etc.) (27, 46). The fact that millions of patients are on long term oral anticoagulants with, like with vitamin K antagonists (VKA) potential impact on the vasculature (calcification in case of VKA), cannot be overestimated (47). The challenge here is to translate the robust animal data that show that anticoagulant agents reduce atherosclerosis burden (48), to clinically relevant applications, considering human (patient) heterogeneity, including genetic background, sex, comorbidity, and medication use. Individual risk profiles and related biomarker phenotyping will again help to better stratify subsets of patients to better assess the effects of anticoagulant agents in specific patients (49). This may help to begin to understand the impact of anticoagulant medication on atherosclerosis and the potential for curing vascular disease with antithrombotic therapy, making optimal use of the “off target” (or pleiotropic) effects of such agents.

It is pivotal to understand how single proteases act on cells and which receptors are involved. Protease activated receptors (PARs) have been studied for several decades, yet, many questions remain to be answered, for example how one enzyme-like thrombin acts both in protective and offensive directions on cells (50). The intelligent use of specific mutant mice that lack target epitopes in PARs, or the use of modified proteases, will help to unravel the ways that proteases like factor Xa, VIIa, thrombin, or activated protein C signal in cells, not only in the CV system but also in other complex diseases like cancer and neuroinflammatory disorders (51, 52). These mechanistic approaches make intelligent use of expensive, but also increasingly controversial animal models, optimally employing state of the art technology like single cell RNA sequencing and high sensitivity imaging to gain detailed and fundamental new knowledge on complex biologic processes that are still hard to mimic in full *in vitro*.

A related, more translational **sixth challenge** is to make optimal use of the expanding spectrum of “omics” technology. Genomics has been around for a while now, widely applied in CVD areas like hypertension, heart failure, and atherosclerosis. Gradually, new information is emerging related to VTE (53–55). The miRNA's linked to venous thrombosis are also being identified, their properties are being explored and the clinical impact needs to be studied (56, 57). Expectations are high, but as we observed in the genomics era, one must be cautious with high expectations from the “omics” field, since GWAS studies did not produce large numbers of new candidate modifiers of venous thrombosis risk. At the same time, the new knowledge from these large and unbiased searches will reveal the candidate modifiers of thrombosis, but also uncover the networks between coagulation and other modifying systems that will also allow better characterization of the individual at risk of thrombosis. Large-scale consortium-based efforts to unravel human biology and thrombotic pathophysiology in mankind are finally taking off (58).

A **final challenge** is to generate the funding required to address these rather comprehensive goals. Over the past decades, clinical trials in thrombosis were substantially financed by pharmaceutical companies. However, since the registration of a generation of direct oral anticoagulants for the largest indications (AF and VTE), pharma's interest in (large trials with) antithrombotic agents has sharply diminished. This may depend on the fact that the margins for gain in benefit/risk ratio have become smaller and also be because efforts to individually tailor antithrombotic therapies have no immediate financial profit for pharma, while this is of greater interest to diagnostic companies that traditionally do not invest substantially in biomedical research. Unfortunately, governments have become more and more economical in the budgets they spend on biomedical research, without any societal justification. For now, much will rely on the creativity of researchers to work in consortia or other models of multidisciplinary, international research to assemble the best investigators for the complex issues at stake. Research has become a team sport, more than ever before. It is essential to tackle the major challenges in thrombosis with swift and concerted actions.

## Author Contributions

The author confirms being the sole contributor of this work and has approved it for publication.

## Conflict of Interest

HC received funding for research from Bayer and Pfizer, is a consultant to Alveron and stockholder in Coagulation Profile, and is a Principal Investigator on the following consortia sponsored by the Dutch Heart Foundation (CVON RACE-5 and CONTRAST), ZON-MW (Dutch Coalition on Thrombosis in COVID-19), and REG MED XB.

## Footnotes

^1^www.WHO.int
